# Histopathological and immunohistochemical analyses do not indicate timing of prophylactic hysterectomy: a retrospective analysis of endometrial biopsies in Lynch syndrome carriers

**DOI:** 10.1007/s10689-026-00611-x

**Published:** 2026-10-15

**Authors:** Lotte van Leeuwen, Ellis L. Eikenboom, Floris H. Groenendijk, Monique E. van Leerdam, Manon C. W. Spaander, Helena C. van Doorn, Anja Wagner

**Affiliations:** 1 Department of Clinical Genetics, Erasmus MC Cancer Institute, University Medical Center Rotterdam, P.O. Box 2040, 3000 CA Rotterdam, The Netherlands; 2 Department of Medical Oncology, Erasmus MC Cancer Institute, University Medical Center Rotterdam, Rotterdam, The Netherlands; 3 Department of Gastroenterology and Hepatology, Erasmus MC Cancer Institute, University Medical Center Rotterdam, Rotterdam, The Netherlands; 4 Department of Pathology, Erasmus MC Cancer Institute, University Medical Center Rotterdam, Rotterdam, The Netherlands; 5 Department of Gastroenterology and Hepatology, Leiden University Medical Center, Leiden, The Netherlands; 6 Department of Gastrointestinal Oncology, Antoni Van Leeuwenhoek Hospital, Amsterdam, The Netherlands; 7 Department of Gynecology, Erasmus MC Cancer Institute, University Medical Center Rotterdam, Rotterdam, The Netherlands

**Keywords:** Endometrial carcinoma, Lynch syndrome, MMR-proteins, PAX2, PTEN, ARID1A

## Abstract

Women with Lynch syndrome are at increased risk of endometrial cancer. Gynecological surveillance is advised, but also prophylactic hysterectomy is an option. This study aimed to gain more insight in tumorigenesis in Lynch carriers to be able to personalize prophylactic hysterectomy timing. To this aim endometrial cancer surveillance in Lynch syndrome carriers was assessed using data from the Dutch Lynch syndrome database linked to the Dutch National Pathology Databank (PALGA). Eligible participants were Lynch syndrome carriers under surveillance, who underwent hysterectomy and had ≥ 1 endometrial biopsy within five years prior to surgery. Pathology reports, describing the histopathological features of the samples, were collected and, if available, samples were revised by a specialized pathologist. Additionally loss of expression of ARID1A, PTEN, PAX2, and mismatch repair (MMR) proteins was studied. Pathology reports from 32 Lynch syndrome carriers were analyzed: 16 had normal endometrium at hysterectomy, five had hyperplasia without atypia, six had endometrial intra-epithelial neoplasia (EIN), and five had endometrial cancer. Prior to hysterectomy, hyperplasia with or without atypia or endometrial cancer was reported in 3% of biopsies in the histopathological normal group, 26% in endometrial hyperplasia without atypia group, 26% in the EIN group, and 36% in the endometrial cancer group. Upon revision, combined neoplastic development of tissue and loss of protein expression were observed in 12% of the normal group, 36% of endometrial hyperplasia without atypia, 36% of EIN, and 50% of endometrial cancer group. So, increasing tissue disorganization and loss of protein expression appeared to correlate with progression to (pre)malignancy. However, no specific marker or consistent pattern emerged that could guide clinical decisions on prophylactic hysterectomy for individual patients. These findings highlight the complexity of endometrial cancer development in Lynch syndrome and underscore the need for further research to improve surveillance strategies and optimize timing of prophylactic surgery.

**Supplementary Information:**

The online version contains supplementary material available at https://doi.org/10.1007/s10689-026-00611-x.

## Introduction

Lynch Syndrome is an autosomal dominant genetic predisposition for the development of a wide variety of cancers. It is not only one of the first identified cancer-syndromes, but also the most common hereditary cancer syndrome worldwide [1]. Lynch Syndrome is caused by a pathogenic germline variant (PV) in one of the DNA mismatch repair (MMR) genes *MLH1*, *MSH2*, *MSH6*, *PMS2*, or due to a deletion of the 3’ end of the *EPCAM* gene [2–6]. It is estimated that Lynch syndrome affects 1 in 279 individuals [7]. Since its initial recognition, Lynch syndrome has been associated with elevated rates of colorectal carcinoma and endometrial carcinoma [8, 9]. Cumulative risks for developing endometrial cancer by age 75 are 37, 48.9, 41.1, and 12.8% for *MLH1*, *MSH2*, *MSH6*, and *PMS2* mutation carriers, respectively [10].

Most research on histological changes during endometrial cancer development has been performed on its most common subtype, endometrioid endometrial cancer. Usually, endometrial cancer is preceded by endometrial hyperplasia: an irregular proliferation of endometrial glands, resulting in glands of varying shapes and sizes [11]. However, endometrial hyperplasia can co-exist with cytological atypia (hyperplasia with atypia also known as endometrial intraepithelial neoplasia, EIN). EIN is thought to be a precursor lesion to endometrial carcinoma, specifically of the endometrioid histological subtype. While it does not necessarily lead to malignancy, approximately 30% of women with EIN will have subsequent endometrial carcinoma [12]. EIN shares molecular genetic alterations that are seen in endometrial carcinoma in the context of Lynch syndrome, such as loss of expression of PTEN or MMR deficiency (MMRd) [13–15]. Hyperplasia, both with and without atypia, has been observed as background in endometrial carcinoma, further corroborating the hypothesis EIN is a precursor lesion [16].

There are several genes thought to be involved in the development of endometrioid endometrial carcinoma, the most common subtype of Lynch syndrome-associated endometrial carcinoma. Common genes implicated in endometroid endometrial carcinoma development are *ARID1A* and *PTEN*, which lose their tumor-suppressor role when mutated, and Raffone et al. have recently shown in different studies that PAX2 protein expression seems to be absent as well [17–20]. As shown by Niskakoski et al. [21], ARID1A loss of expression coincides with increasing histopathological changes. Additionally, a loss of MMR-protein expression is seen in all Lynch syndrome-associated cancers—including endometrial cancer. However, MMRd can already be observed in non-neoplastic endometrial glands, which also indicates a higher risk for development of subsequent EIN and/or endometrial carcinoma, as 53% of EC patients have previous MMRd [22–24]. Clonal loss of MMR protein expression may therefore serve as a reliable biomarker to predict risk for endometrial cancer development in Lynch syndrome carriers. In addition, microsatellite instability has been shown to be a reliable biomarker together with MMRd, indicative of early (pre)malignant development in the endometrium [25].

Although several genes implicated in endometrial cancer development have been identified, information is lacking on the pathway(s) involved in each morphological stage towards endometrial cancer development in female Lynch syndrome carriers. As these patients are at an increased risk for the development of endometrial carcinoma, understanding carcinogenesis may help to define effective preventive measures and their timing. The aim of this study was to provide insight into this process by reviewing consecutive endometrial biopsies and hysterectomy specimens during gynecological surveillance of female Lynch syndrome carriers. Original pathology reports were collected and analyzed, in addition to histopathological revisions of biopsies.

## Methods

### Patient selection

In this retrospective cohort study, data from female Lynch syndrome carriers were collected from a nation-wide database of the Foundation on the Detection of Hereditary Tumours (Stichting Opsporing Erfelijke Tumoren, or StOET) in Leiden. The StOET is a nation-wide registry for hereditary cancer syndromes, such as Lynch Syndrome. The study was approved by the Erasmus MC Medical Ethics Committee (MEC-2020-0600).

**Fig. 1:**
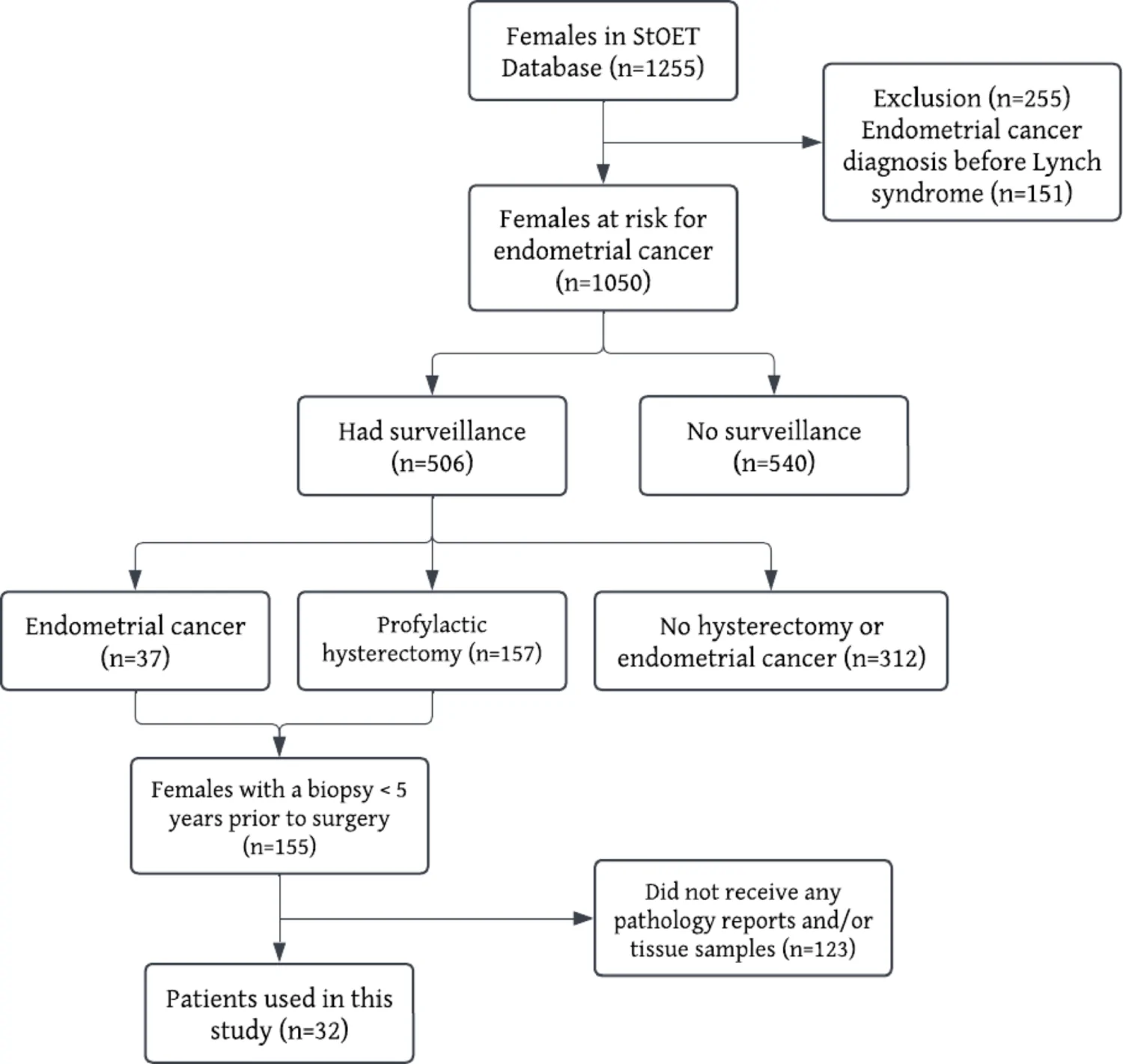
Flowchart depicting the patient selection process

Figure 1 illustrates the patient selection for this study. Firstly, 1225 female Lynch syndrome carriers registered in the database at 22nd of February 2022 were identified. Secondly, those who had gynecological surveillance were selected via the StOET database. Only those women with a hysterectomy were included, either as prophylactic measure or after endometrial carcinoma diagnosis.

### Data and sample selection and work-up

After patient selection, corresponding patient files from the Dutch Nationwide Pathology Databank (PALGA) were linked via secured linking procedure to ensure a complete overview of performed biopsies and hysterectomies in these patients. Patients with a hysterectomy and at least one endometrial biopsy within 5 years prior to hysterectomy were included, to obtain protein expression patterns over time.

The hysterectomy sample was used as an outcome measure, to define four different groups for comparison, (1) women with normal endometrium at hysterectomy, further indicated as the normal group, (2) women with endometrial hyperplasia without atypia at hysterectomy, (3) women with EIN at hysterectomy and (4) women with endometrial cancer at hysterectomy. All retrieved sample reports and tissue specimens were (re-)classified and analyzed according to the 2014 WHO classification.

Demographics of patients, including age at biopsy and/or hysterectomy, number of biopsies, interval between biopsies, and specific MMR mutation were obtained from patient files and pathology reports. Clinical data on the menstrual cycle was available and used for interpretation of results from both the original pathology reports and the revisions.

A dedicated gyno-pathologist revised all retrieved specimen, while blinded for the original pathology report. The received tissues were from the same fixated tissue block used by the original pathologist. First, the tissue was sliced and stained for hematoxylin and eosin and assessed for presence of possible hyperplasia with or without atypia, or endometrial cancer. Tissue samples were considered representative if 10 or more endometrial glands were present. These findings were afterwards correlated with the original pathology report to inspect inter-observer reliability of histopathological analysis. In case of discrepancy with the original report, the review was assessed with a second pathologist.

Second, samples were stained by immunohistochemistry (IHC) for ARID1A, PTEN, PAX2 and the four DNA MMR proteins (MLH1, MSH2, MSH6, and PMS2). IHC staining was performed in non-neoplastic tissue, and if present, in the (pre)malignant lesions by automated IHC using the Ventana Benchmark ULTRA (Ventana Medical Systems Inc.). Sequential 4 µm thick (FFPE) sections were stained using Optiview detection (OV) (#760–700, Ventana). In brief, following deparaffinization and heat-induced antigen retrieval with CC1 (#950–500, Ventana), the tissue samples were incubated with antibody of interest for the indicated time (see for details supplementary Table 1). Incubation was followed OV detection and hematoxylin II counter stained for 8 min followed by a blue coloring reagent for 8 min according to the manufactures instructions (Ventana).

### Statistical analysis

Due to the small sample size and the descriptive nature of the study, no statistical analyses were conducted.

### Data availability statement

Raw data is not publicly available to preserve individuals’ privacy under the European General Data Protection Regulation. Anonymized histological and pathological data is available upon request for independent additional analysis for the reproducibility of this study in other centers.

## Results

We received a total of 181 requested original pathology reports of 32 patients, and 85 corresponding samples were available for revision. Of the pathology reports, 149 were from endometrial biopsies and 32 were from the hysterectomy sample. Based on the original diagnosis at hysterectomy we identified 16 Lynch syndrome carriers with normal endometrium, 5 with hyperplasia without atypia, 6 with EIN, and 5 with endometrial cancer. All 5 endometrial carcinomas were found in TNM-stage T1aN0M0 or FIGO stage 1A and of endometrioid histology. Three endometrial carcinomas were grade 2, while the two others were grade 1. The original pathology reports only mentioned data on histopathology, but not on immunohistochemistry of our proteins of interest. Of the tissue samples available for revision, 15 were from hysterectomies and 70 were from endometrial biopsies (e.g. pipelle or curettage). Baseline characteristics are given in Table 1.

**Table 1:** Patient characteristics

	Normal	Hyperplasia without atypia	EIN	Carcinoma	Total
*Collected data*					
Number of females	16	5	6	5	32
Number of original pathology reports of biopsies	66	23	35	25	149
Number of original pathology reports of hysterectomies	16	5	6	5	32
Number of tissue samples of biopsies	25	11	14	20	70
Number of tissue samples from hysterectomies	8	3	2	2	15
*Characteristics*					
Age at first biopsy, median [IQR]	40.5 [38.25–45.75]	35 [34–55.5]	43 [43– 45]	41 [38–46]	41 [38–46.5]
Age at hysterectomy, median [IQR]	45.5 [41.25–52.5]	51 [38.5–60]	52 [45–52]	45 [41.5–53]	47.5 [41.25–52]
Surveillance period in months, median [IQR]	60 [17–96.25]	53 [51–120.5]	72 [35–178]	43 [26.5–89.5]	54.5 [24.25–96.25]
Mean number of biopsies [range]	4.1 [1–12]	4.6 [2–6]	5.8 [3–14]	5 [2–9]	4.7 [1–14]
*Gene of MMR pathogenic variant*					
MLH1	4	1	0	2	7
MSH2	3	4	5	3	15
MSH6	7	0	1	0	8
PMS2	2	0	0	0	2

**Fig. 2:**
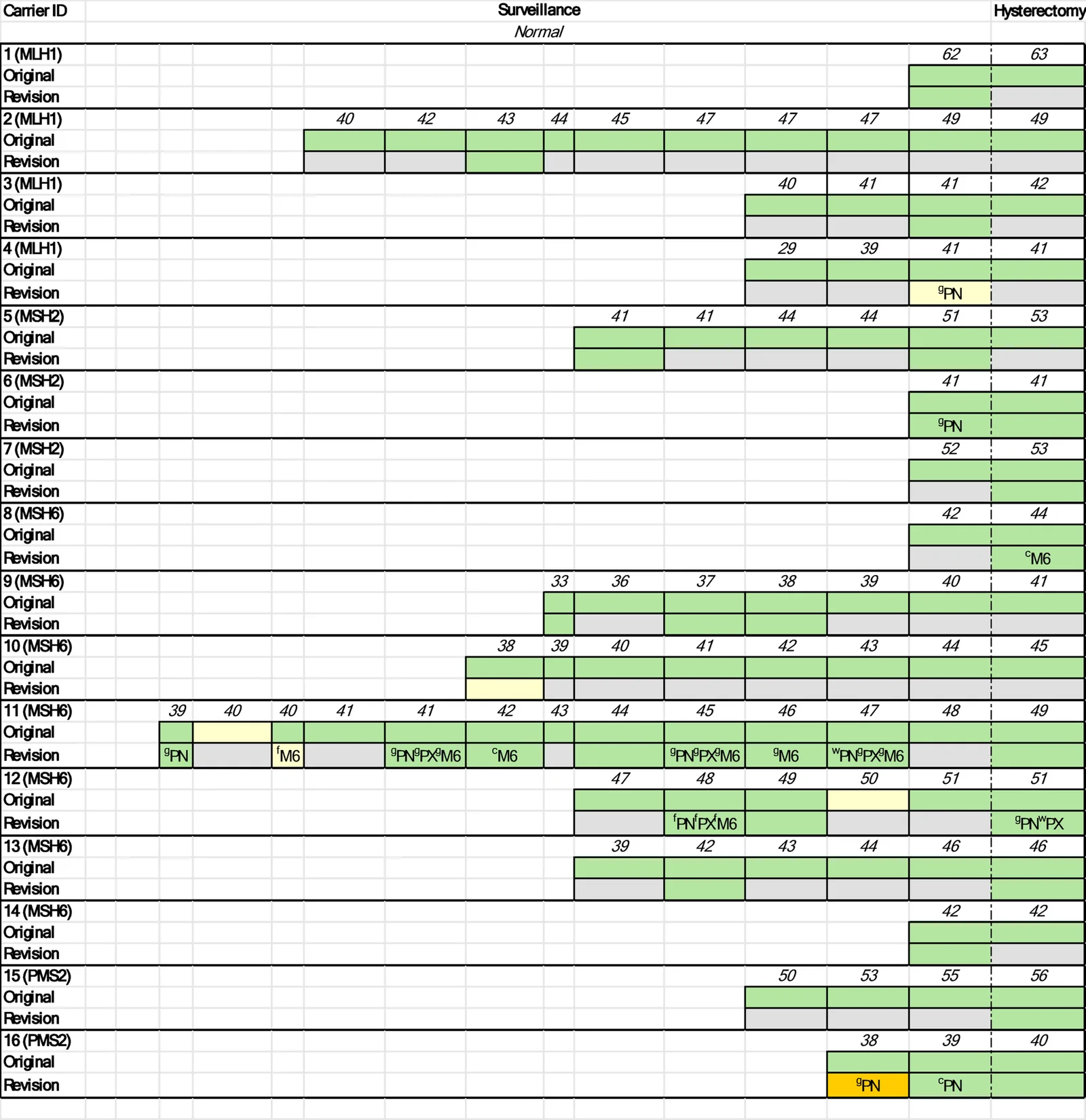

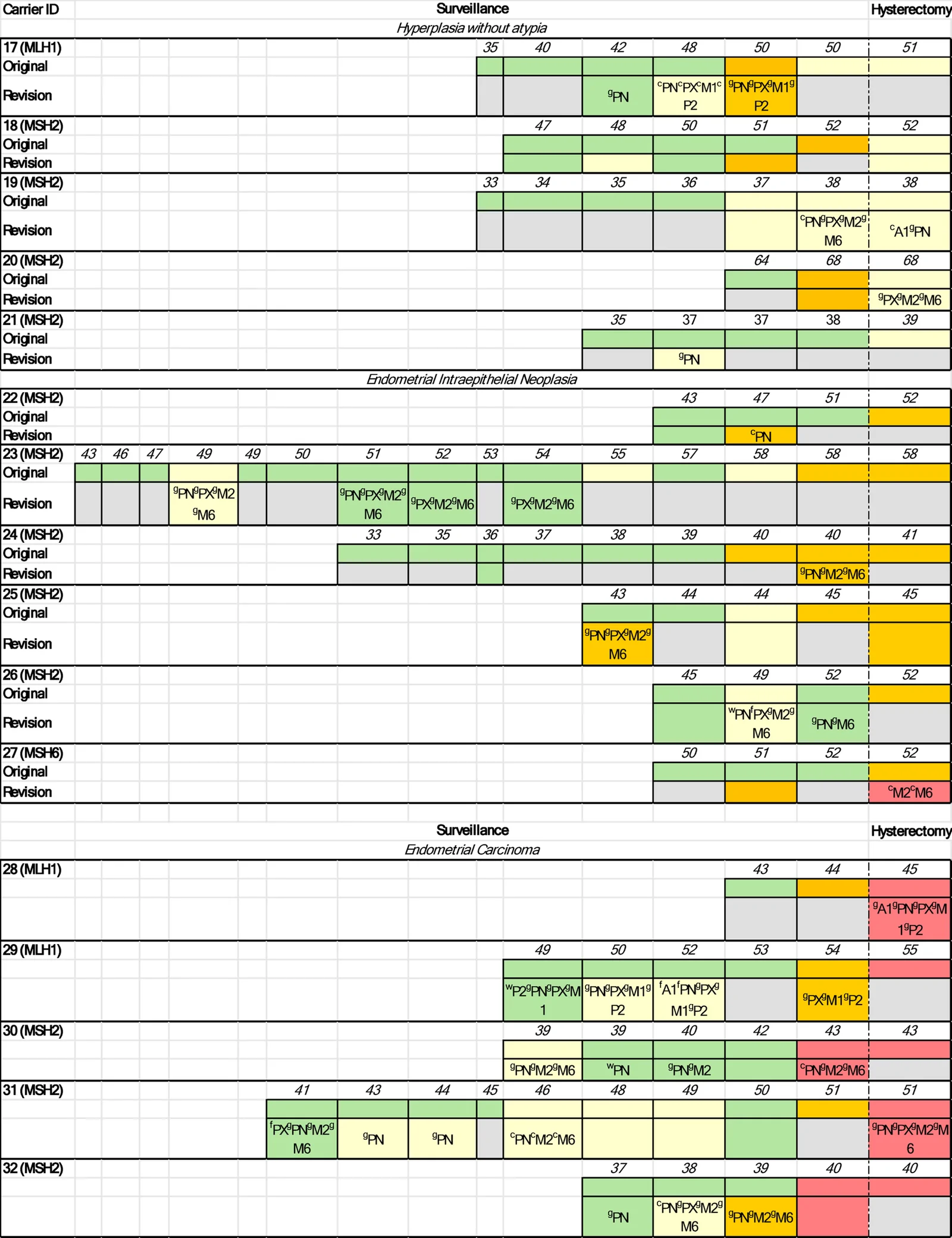

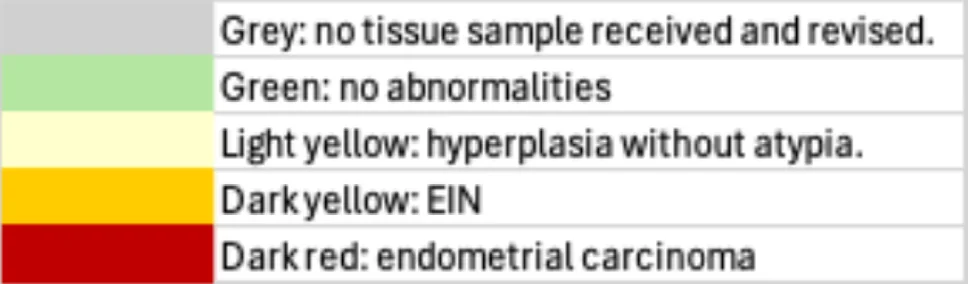
Overview of biopsy revisions in the different groups indicating neoplastic development and loss of protein expression, with the last cell indicating the hysterectomy sample (right of the dotted line). Specific protein loss has been indicated in the cell, together with the expression loss. Proteins not mentioned in the revisions indicate normal expression. Age at each biopsy/hysterectomy is given in the first row per patient in italics. A1: ARID1A, PN: PTEN, PX: PAX2, M1: MLH1, M2: MSH2, M6: MSH6, P2: PMS2. ^g^general loss of expression. ^c^(sub)clonal loss of expression. ^f^focal loss of expression. ^w^weak expression

Figure 2 shows the longitudinal analysis of both the original pathology reports and their corresponding revisions. In the normal group (i.e. with normal endometrium at hysterectomy), a total of 66 original pathology reports were collected from biopsies before hysterectomy. Two reports (3%) described endometrial hyperplasia without atypia, and 64 normal histology (97%). A total of 23 original pathology reports from biopsies were collected from female LS-carriers with hyperplasia without atypia at hysterectomy. In this group, six reports (26%) described pathological tissue changes in the biopsy, three reports described hyperplasia without atypia (13%), and three reported EIN (13%). In thirty-five original pathology reports collected of those with EIN at hysterectomy, (pre)malignant tissue changes were found in nine biopsies (26%) as reported by the original pathologist. Five biopsies showed hyperplasia without concurrent atypia (15%) and 4 could be classified as EIN (11%). In females with endometrial carcinoma at hysterectomy, 25 pathology reports from biopsies before hysterectomy were collected. Based on these reports, nine biopsies showed (pre)malignancy (36%). Seven reports described endometrial hyperplasia, of which four occurred without atypia (16%) and three were EIN (8%) while two reports described endometrial carcinoma in the biopsy preceding hysterectomy (12%).

Of 25 tissue samples collected of biopsies before hysterectomy in the normal group, four (16%) were classified by the revising pathologist as having abnormal histology, three as endometrial hyperplasia without atypia (12%) and one other as EIN (4%). Another 11 tissue samples from biopsies before hysterectomy were collected in the group with endometrial hyperplasia without atypia at hysterectomy. Of these, eight samples had a abnormal histology upon revision (73%). Five biopsies showed hyperplasia without atypia (46%), and three were classified as having EIN (27%). In the group of females with EIN at hysterectomy, 14 tissue specimens of biopsies prior to hysterectomy were collected. Based on revisions, half of these biopsies showed (pre)malignancy (50%). Three biopsies showed signs of hyperplasia without atypia (21%), and four others showed EIN (29%). In the group of females with endometrial carcinoma at time of hysterectomy, 20 biopsies were collected before hysterectomy. Of these revised biopsies, 13 showed abnormal histology (65%). Nine biopsies showed endometrial hyperplasia without atypia (45%), while two others displayed EIN (10%). Two biopsies showed endometrial carcinoma (10%); these were both taken shortly before hysterectomy.

Of the in total 85 revisions of samples taken before and during hysterectomy 29/33 (88%) were in accordance with the original pathology report in the normal group, 9/14 (64%) in the hyperplasia without atypia group, 9/16 (56%) in the EIN group, and 11/22 (50%) in the endometrial cancer group. So overall, there was a concordance of 68% (58/85).

Of the 25 revised biopsies before hysterectomy in the normal group, 12 (48%) biopsies depicted loss of protein expression. While ARID1A had normal staining amongst all samples, PTEN showed loss of expression in nine biopsies (36%). Six of these showed a general loss of PTEN, while weak staining, focal, and subclonal loss was found in one biopsy each. There was loss of PAX2 expression in four biopsies (20%); three showed a general loss of expression and one focal loss. Of the MMR-proteins, only MSH6 showed loss of expression in seven biopsies (28%) in two *MSH6*-PV carriers. Four biopsies had a general loss of expression of MSH6, two focal, and one subclonal loss of expression.

**Fig. 3:**
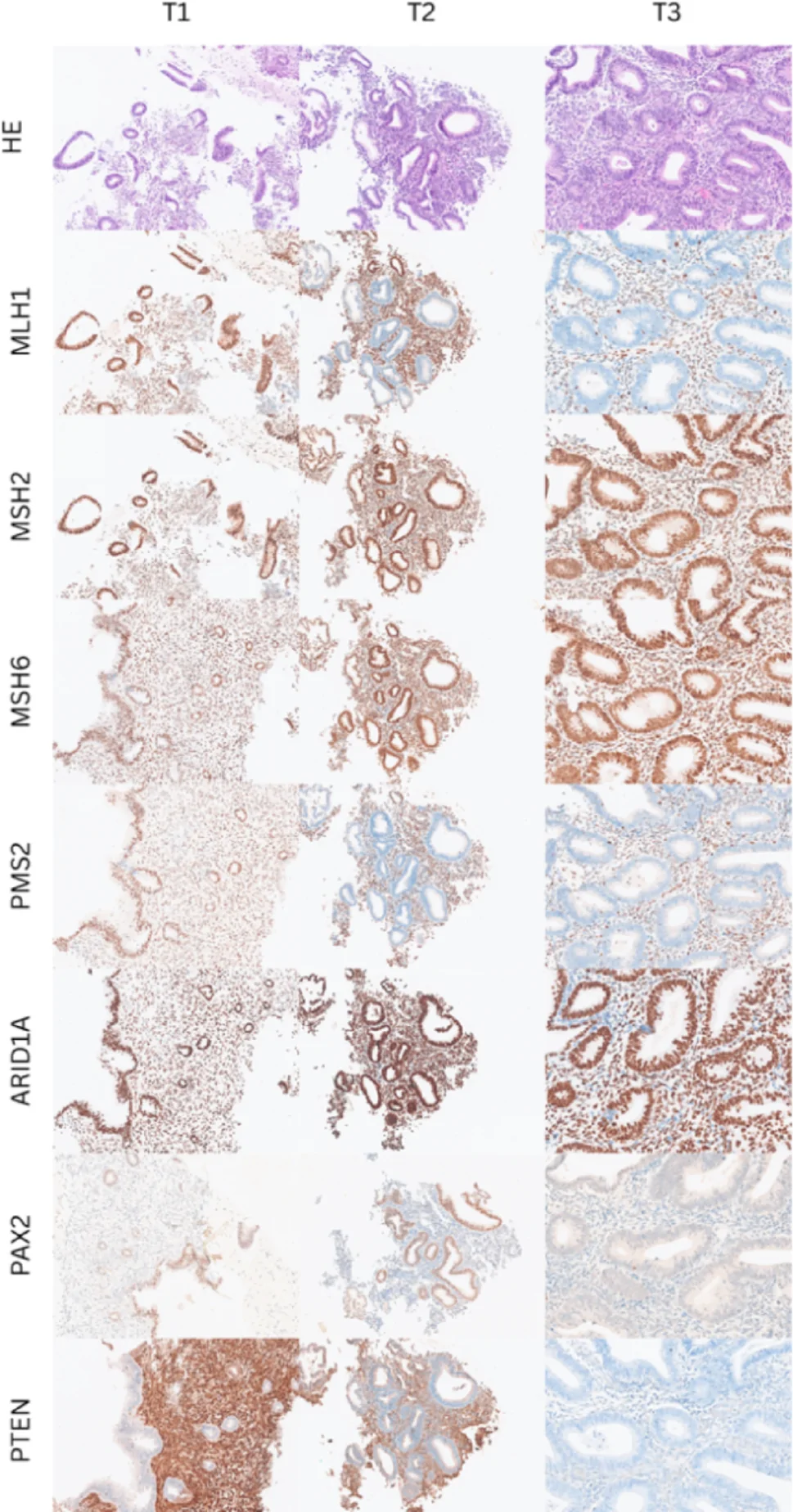
Overview of protein expression over time in patient 17, who has a PV in MLH1. These are three subsequent biopsies taken within the span of 40 months. T1: PTEN loss of expression in <5%. T2: PTEN subclonal loss of expression in 20%; PAX2 subclonal loss of expression in 5%; MLH1 subclonal loss of expression in 5%; PMS2 subclonal loss of expression in 5%. T3: PTEN loss in 100%, PAX2 loss in 15%, MLH1 loss in 15%, PMS2 loss in 15%

All 11 biopsies before hysterectomy in carriers with endometrial hyperplasia without atypia at hysterectomy showed normal ARID1A expression. PTEN expression was lost in five (46%) biopsies. Of these five, two biopsies showed a (sub)clonal loss, and three general loss of expression. In three biopsies, loss of PAX2 expression was observed (27%); one biopsy showed a (sub)clonal loss and the two other biopsies a general loss of PAX2. Loss of expression in MMR-protein(s) were seen in three biopsies (27%); one subclonal and one general loss of MLH1 and PMS2 in a *MLH1*-PV carrier, and one general loss of MSH2 and MSH6 expression in a *MSH2*-PV carrier. An example of protein loss detected by IHC in a *MLH1*-PV carrier is shown in Fig. 3.

In the 14 biopsies received for revision in the EIN group, none showed loss of ARID1A expression. PTEN loss of expression was seen in seven biopsies (50%), of which one was a (sub)clonal loss, one a weak expression, and five were general losses. Loss of PAX2 expression was seen in six biopsies (43%), of which one was a focal loss and five others were generalized. In eight biopsies a loss of one of the MMR-proteins was seen (57%). One *MSH2*-PV carrier had general loss of MSH2 and MSH6 in four biopsies. MSH2 and MSH6 loss was observed in one biopsy of three other different *MSH2*-PV carriers.

Of 20 revised biopsies before hysterectomy in the group of female Lynch syndrome carriers with endometrial carcinoma at hysterectomy, 15 (75%) showed loss of expression of at least one protein. The only loss of ARID1A expression was observed in this group, concerning focal loss of expression in one biopsy (4%). PTEN expression was lost in fourteen biopsies (70%), of which one biopsy showed a weak expression pattern, one a focal loss, two a subclonal loss, one a clonal loss, and nine biopsies a general loss of PTEN expression. Loss of PAX2 expression was found in six biopsies (30%), of which one a focal and five a general loss. MMR-protein loss of expression was found in eleven revised biopsies (55%). General loss of MLH1 expression was seen in combination with PMS2 loss in four biopsies in a *MLH1*-PV carrier. MSH2 and MSH6 loss was seen in six biopsies, of which one subclonal, and one single general loss of MSH2 in three *MSH2*-PV carriers.

Overall, 41 of 70 revised biopsies (59%) showed loss of expression in at least one protein. In the normal group, 12 out of 25 revisions showed loss of protein expression (48%), in the endometrial hyperplasia without atypia group 5 out of 11 biopsies (46%), in the EIN group 9 out of 14 biopsies (64%), and in the endometrial cancer group 15 out of 20 biopsies (75%). The loss of protein expression was predominantly seen in PTEN, PAX2 and MMR-proteins, the latter corresponding with the germline MMR-gene-PV.

Neoplastic tissue evolvement and/or loss of protein expression of ARID1A, PTEN, PAX2 and/or MMR proteins were found in 13/25 (52%) biopsies prior to hysterectomy in the normal group, in 9/11 (82%) biopsies in the endometrial hyperplasia without atypia group, in 11/14 (79%) biopsies in the EIN group, and in 18/20 (90%) biopsies in the endometrial cancer group, respectively. A combination of neoplastic tissue development and loss of protein expression were seen in 3/25 (12%) in the normal group, 4/11 (36%) in the endometrial hyperplasia without atypia group, 5/14 (36%) in the EIN group, and 10/20 (50%) in the endometrial cancer group.

## Discussion

The aim of this retrospective cohort study was to gain insight into the tumor development of endometrial cancer in female Lynch syndrome carriers to optimize timing of prophylactic hysterectomy in carriers under gynecological surveillance. Our results show that however neoplastic tissue development and loss of protein expression seem to correlate with development toward (pre)malignancy, no specific marker or consistent pattern emerged that could guide clinical decisions on prophylactic hysterectomy for individual patients.

The analyses of 149 original pathology reports of endometrial biopsies taken during surveillance of Lynch syndrome carriers and the revision of 70 corresponding biopsies did show an increase of histopathological abnormalities comparing women with normal histology, hyperplasia without atypia, EIN and endometrial cancer at hysterectomy respectively. However, there was no consistent progression from normal endometrium to malignancy. In two cases (18 and 20) regression from EIN to hyperplasia without atypia is seen and the sample prior to hysterectomy in an endometrial cancer case (30) does not display any abnormalities. So, while the presence of EIN in a surveillance biopsy seems a valid indication of increased risk of developing malignancy and thus for clinical intervention, it may lead to premature or delayed hysterectomy in individual cases.

Regarding the immunohistochemical analyses, loss of ARID1A expression was found in one of the two stained endometrial carcinomas in this study. In literature, this is reported in up to 40–54% of endometrioid endometrial carcinoma cases and in EIN [20, 26]. However, in biopsies prior to endometrial carcinoma we only found loss of ARID1A expression in one case (29), questioning its value in indicating early endometrial cancer development. The ARID1A loss did co-localize with concurrent MMRd and PTENd, which is a pattern also observed by Canet-Hermida et al. in normal endometrial glands in women with Lynch syndrome [25]. We observed this ARID1A loss in hyperplasia, possibly underlining their hypothesis that ARID1A loss of expression is an early driver in endometrial carcinoma development.

Apart from expression loss in neoplastic tissue, PTEN and MMR expression can be lost in non-neoplastic endometrial tissue as well. Earlier studies reported that PTEN loss of expression can be seen in up to 20% of non-neoplastic endometrial samples of women without Lynch syndrome [27]. MMR-loss of expression was seen in 70% of normal endometrial tissue in women with Lynch syndrome [22]. Kauppinen et al. have also indicated that multiple molecular changes can be present already in normal endometrium before (pre)malignant development, like the loss of MMR expression[15]. We have shown in addition to these studies that even in normal endometrium of women, who did not develop endometrial (pre)malignancies, these molecular changes can be present. As expected, MMR-protein expression loss in this study corresponded with the germline mutation. While MMRd is thought to mark the start of endometrial neoplastic development in women with Lynch syndrome [28], we found multiple cases with isolated PTEN expression loss preceding MMRd. However, this is not enough data to make any assumptions about the possible molecular pathways in endometrial tumorigenesis.

In this study, loss of protein expression seems to increase over time as neoplastic tissue develops (see Fig. 2). These same patterns have been reported by Niskakoski et al. in the context of Lynch syndrome, where loss of MMR-expression increases as the endometrial cells become neoplastic [21]. However, protein expression loss does not seem to predict development of EIN or endometrial carcinoma. In the carriers with either endometrial hyperplasia without atypia, EIN or endometrial cancer at hysterectomy, loss of protein expression preceded neoplastic development only in four cases (25%). The same phenomenon has been described by Kloor et al. in MMRd intestinal crypt foci, concluding that MMRd does not necessarily lead to cancer [29]. More often than loss of protein expression being the first apparent abnormality, a combination of neoplastic development and loss of expression in one or more proteins is seen (38% of cases). This challenges the diagnostic usefulness of IHC staining during the surveillance period as an addition to analyzing morphological abnormalities in tissue histology.

When looking at histopathological, molecular and a combination of abnormalities, there is an observable increase in molecular alterations correlated with morphological tissue status at time of hysterectomy. However, even when a combination of the above-mentioned features is used as a criterion for prophylactic hysterectomy in individual patients, this does not guarantee correct timing of the hysterectomy.

Notwithstanding the neoplastic morphological and immunohistochemical changes, there seem to be outliers in the surveillance period. In many cases, an abnormal biopsy is followed by a normal biopsy and vice versa. There does not seem to be a clear-cut development of pathological abnormalities during the surveillance period. This can be due to two different phenomena: (1) There is a so-called ‘sampling error’; a woman has hyperplasia, but during the biopsy a small part without this endometrial hyperplasia is taken and the hyperplasia will thus not be found during biopsy. (2) This alleged regression may also be due to the immunological nature of Lynch syndrome-associated neoplasms [30]. This could explain why certain lesions and loss of protein expression could resolve over time. It has been suggested the same happens in Lynch syndrome-associated colorectal lesions [31].

Using neoplastic development only, loss of protein expression only, or the combination of the two as criterion for prophylactic hysterectomy will regularly give female Lynch syndrome carriers a hysterectomy too soon or too late. Presently, there is no evidence to implement IHC analysis into clinical practice, and usefulness of gynecological surveillance via endometrial biopsy remains debatable. Further studies into the carcinogenesis of Lynch syndrome-associated endometrial carcinoma are needed to provide accurate and reliable advice about prophylactic procedures in this context.

Unfortunately, this study also has limitations. Firstly, it has a small sample size. This may not give a full overview of what is really happening in the population. The small sample size was both due to the small number of women with Lynch syndrome and a (prophylactic) hysterectomy in combination with one biopsy within five years prior to surgery and to receiving fewer pathology reports and tissues from fewer patients than originally requested. The limited amount of tissue available at biopsy or the exhaustion of the amount of tissue still available due to earlier studies might be a reason why we received so little tissue for revision.

Another point of concern is the inter-observer concordance, which was 68% in this study. The original pathologist and our revising pathologist had different conclusions on multiple occasions, especially when analyzing seemingly abnormal endometrial tissue. Inter-observer concordance in endometrial hyperplasia has been shown to be a hurdle in multiple studies, especially due to analysis ‘styles’ of different pathologists, i.e. the tendency to either over- or underdiagnose [32]. The poor inter-observer concordance makes it hard to reliably diagnose patients and screen female Lynch syndrome carriers over time, in addition to the possibility of a sampling-error occurring within one tissue-slide.

Notwithstanding these limitations, this study is unique, as pathology reports and tissue specimens have been collected in female Lynch syndrome carriers with and without EIN or endometrial carcinoma at prophylactic hysterectomy. The data from this group gives better insight into which molecular alterations are EIN and endometrial carcinoma-specific, and which can be found in non-neoplastic tissue as well. To our knowledge, this is the first study investigating Lynch syndrome-related endometrial carcinogenesis during the complete surveillance period and using a cohort with nation-wide coverage. Linking this StOET database to the PALGA database ensures an accurate overview of gynecological surveillance per patient. Also, when available, samples were reviewed by a specialized pathologist and additional immunohistochemical analyses were performed.

## Conclusion

To conclude, this study gives insight into the histopathological and immunohistochemical changes during the gynecological surveillance period of female Lynch syndrome carriers. While histopathological and molecular changes seem to increase as (pre)malignancy develops, no specific marker or consistent pattern emerged that can be used for timing of prophylactic hysterectomy in individual patients. Large prospective cohort studies are needed to elucidate how optimal timing for prophylactic hysterectomy in female Lynch syndrome carriers can be achieved.

## Supplementary Information

Below is the link to the electronic supplementary material.

Supplementary Material 1

## Data Availability

Raw data is not publicly available to preserve individuals’ privacy under the European General Data Protection Regulation. Anonymized histological and pathological data is available upon request for independent additional analysis for the reproducibility of this study in other centers.
