# Supplementary material for: Histopathological and immunohistochemical analyses do not indicate timing of prophylactic hysterectomy: a retrospective analysis of endometrial biopsies in Lynch syndrome carriers
Source: Fam Cancer. 2026 Oct 15;25(4):95. doi: 10.1007/s10689-026-00611-x (PMC13672446; doi:10.1007/s10689-026-00611-x)
Supplement: Supplementary file 1 [file 10689_2026_611_MOESM1_ESM.docx]

Supplementary Table 1. Information on the used antibodies and procedure

| Antibody | Dilution | Species | Company | Clone | Pretreatment  in minutes | Ab incubation  time at 37˚C |
| --- | --- | --- | --- | --- | --- | --- |
| PAX2 | 153 µg/ml | Rabbit | Bio-SB | EP235 | CC1 76’ OV | 40 minutes |
| PTEN | 3,0 µg/ml | Rabbit | Ventana | SP218 | CC1 64’ OV | 32 minutes |
| ARID1A | 1/1200 | Rabbit | Abcam | EPR13501 | CC1 32’ OV | 32 minutes |
| MSH2 | 20 µg/ml | mouse | Ventana | G219-1129 | CC1 40’ OV | 8 minutes |
| MLH1 | 1/40 | mouse | Leica | NCL-L-MLH1 | CC1 40’ OV | 60 minutes |
| PMS2 | 1/200 | rabbit | Bio-SB | EP51 | CC1 56’ OV | 32 minutes |
| MSH6 | 1 µg/ml | rabbit | Ventana | SP93 | CC1 64’ OV | 8 minutes |
